# Increased Collagen Crosslinking in Stiff Clubfoot Tissue: Implications for the Improvement of Therapeutic Strategies

**DOI:** 10.3390/ijms222111903

**Published:** 2021-11-02

**Authors:** Jarmila Knitlova, Martina Doubkova, Adam Eckhardt, Martin Ostadal, Jana Musilkova, Lucie Bacakova, Tomas Novotny

**Affiliations:** 1 Institute of Physiology of the Czech Academy of Sciences, Videnska 1083, 142 20 Prague, Czech Republic; jarmila.knitlova@fgu.cas.cz (J.K.); adam.eckhardt@fgu.cas.cz (A.E.); jana.musilkova@fgu.cas.cz (J.M.); lucie.bacakova@fgu.cas.cz (L.B.); 2 Second Faculty of Medicine, Charles University, V Uvalu 84, 150 06 Prague, Czech Republic; 3 Department of Orthopaedics, First Faculty of Medicine, Charles University and Na Bulovce Hospital, Budinova 2, 180 81 Prague, Czech Republic; martinostadal@yahoo.com; 4 Department of Orthopaedics, University J.E. Purkinje and Masaryk Hospital, 401 13 Usti nad Labem, Czech Republic; tomas.novotny@kzcr.eu; 5 Department of Histology and Embryology, Second Faculty of Medicine, Charles University, 150 06 Prague, Czech Republic

**Keywords:** relapsed clubfoot, congenital idiopathic *Talipes equinovarus*, collagen, contraction, crosslinking, beta-aminopropionitrile (BAPN), fibrosis

## Abstract

Congenital clubfoot is a complex musculoskeletal deformity, in which a stiff, contracted tissue forms in the medial part of the foot. Fibrotic changes are associated with increased collagen deposition and lysyl oxidase (LOX)-mediated crosslinking, which impair collagen degradation and increase the tissue stiffness. First, we studied collagen deposition, as well as the expression of collagen and the amount of pyridinoline and deoxypyridinoline crosslinks in the tissue of relapsed clubfoot by immunohistochemistry, real-time PCR, and enzyme-linked immunosorbent assay (ELISA). We then isolated fibroblast-like cells from the contracted tissue to study the potential inhibition of these processes in vitro. We assessed the effects of a LOX inhibitor, β-aminopropionitrile (BAPN), on the cells by a hydroxyproline assay, ELISA, and Second Harmonic Generation imaging. We also evaluated the cell-mediated contraction of extracellular matrix in 3D cell-populated collagen gels. For the first time, we have confirmed significantly increased crosslinking and excessive collagen type I deposition in the clubfoot-contracted tissue. We successfully reduced these processes in vitro in a dose-dependent manner with 10–40 µg/mL of BAPN, and we observed an increasing trend in the inhibition of the cell-mediated contraction of collagen gels. The in vitro inhibitory effects indicate that BAPN has good potential for the treatment of relapsed and resistant clubfeet.

## 1. Introduction

Idiopathic *Talipes equinovarus* (TEV, clubfoot) is an isolated congenital defect of a lower limb, which occurs with prevalence of 1 or 2 cases per 1000 births [1]. It affects the bony, muscular, and ligamentous structures in one or both feet. The genetic background of clubfoot, and also the effects of various environmental factors on its occurrence, have led researchers to the conclusion that its etiology is probably multifactorial [2,3]. Although the deformity is well recognizable at birth by its morphologic severity, its stiffness can vary greatly—from mild stiffness to an extremely rigid foot that is resistant to manipulation [4]. The Ponseti method, which utilizes serial manipulation and corrective casting, is the most frequent treatment of clubfoot worldwide [5]. Despite its high effectiveness in primary correction, relapses are common, especially if the patient’s parents do not adhere strictly to the regimen [6,7,8,9]. If conservative treatment is repeatedly unsuccessful, surgical release may be considered to achieve correction [8], albeit with the risk of possible long-term, post-operative complications [4].

The mass of pathological stiff tissue localized between the medial malleolus, sustentaculum tali, and navicular bone, characteristic for clubfoot, contributes to maintaining the deformity [10,11]. Several studies have observed the presence of myofibroblast-like cells, known to cause contractile changes of extracellular matrix (ECM) [11,12,13], and have reported an overexpression of collagen type I and III [14] in medial clubfoot tissue. In contrast, a study by Khan et al. [15] described the absence of typical myofibroblast cells. Research carried out by our group has revealed increased levels of fibrosis-associated proteins (collagen type III, V, and VI, transforming growth factor beta, transforming growth factor-inducible protein, tenascin-C, and asporin) [16,17] and increased angiogenesis in the contracted medial tissue of relapsed patients [18].

Fibrosis is characterized by the overdeposition of insoluble fibrillary collagen type I due to an increase in its production and/or a simultaneous decrease in its degradation. The quality and the quantity of crosslinks formed during collagen biosynthesis are one of the important factors determining the properties of ECM. The increase in collagen crosslinking leads to a stiffer collagen network [19]. The formation of ECM that is more resistant to degradation and turnover could be an important factor maintaining the deformity.

To date, only a small number of studies have investigated the possibility of antifibrotic treatment for clubfoot by blocking profibrotic pathways [14,20]; however, no follow-up has been reported. We explored the idea of targeting post-translational modifications of procollagen chains, and we obtained promising results with lysyl hydroxylase inhibition [21]. Post-translational modifications of collagen affect the way and the quantity in which crosslinks are formed in the newly-created collagen fibers. In the present study, we focus on another group of crosslinking-mediating enzymes that are involved in tissue repair and fibrosis, namely lysyl oxidases (LOXs). These enzymes are responsible for the formation of allysine or hydroxyallysine aldehydes, and they catalyze covalent crosslinking of collagen and elastin fibers. Crosslinking mediated by LOXs and by LOX-like proteins (LOXLs) contributes to the stiffness of ECM and of the tissue in general (reviewed in [22]). 

Beta-aminopropionitrile (BAPN) specifically and irreversibly inhibits the activity of LOXs and LOXLs by targeting their active site. BAPN has been reported to reduce the formation of insoluble collagen with minimal cytotoxicity in vitro [23]. BAPN has also been successfully tested in vitro and in vivo for prevention of scarring [24,25], and in post-infarction recovery [26].

We hypothesize that the stiffness of the fibrotic tissue in clubfoot may be closely associated with increased collagen crosslinking. In this study, we assess the extent of collagen expression and production together with the level of crosslinks in the stiff medial part of clubfoot tissue, and we also evaluate the anti-fibrotic effect of BAPN in vitro in cultures of clubfoot-derived cells. The inhibition of crosslinking and deposition of collagen could have a potential for future adjunctive treatment of clubfoot. The controlled reduction of the tissue contraction could facilitate the Ponseti treatment regimen in resistant and rigid clubfeet, and could improve the outcome for relapsed patients.

## 2. Results

### 2.1. Quantitative Changes of Collagen in the Medial Part of Clubfoot

**Figure 1:**
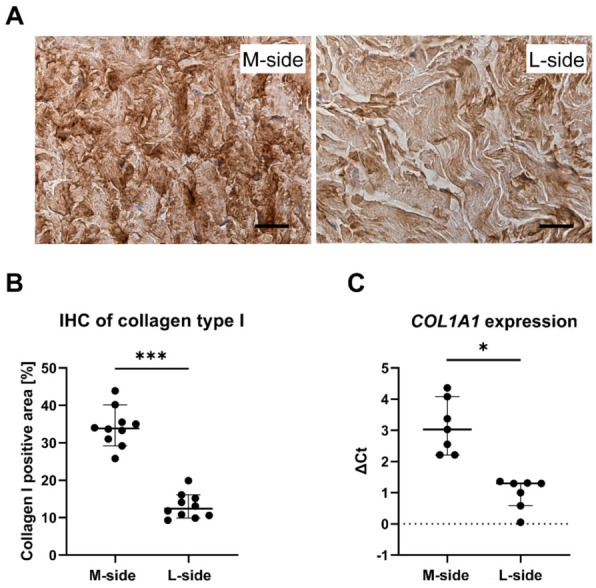
(**A**) Immunohistochemical staining of a dense irregular connective tissue of clubfoot by anti-collagen type I antibody (Scale bar = 20 µm): The pictures illustrate the difference in signal positivity (intensity of brown color) between the M-side and the L-side. The difference in signal positivity was quantified by an image analyzer. (**B**) Quantification of the positively immunostained area. Data are presented as the percentage of the area showing positive immunostaining in M-side and L-side clubfoot tissue. Median with a 95% confidence interval. Paired t-test; statistical significance: *** *p* < 0.0001. (**C**) Relative expression of the *COL1A1* gene (normalized to the *B2M* reference gene) in clubfoot tissue. The data in both graphs are presented as median with a 95% confidence interval. Wilcoxon matched-Paired test; statistical significance: * *p* = 0.0156.

In order to describe local changes in collagen content in relapsed clubfoot, we first assessed the amount of collagen in the contracted medial (M-)side and in the non-contracted lateral (L-)side of clubfoot. Immunohistochemical staining of the tissue slices from both sides revealed a significantly increased amount of collagen type I deposited in the M-side tissue of clubfoot in comparison with the L-side tissue (*n* = 10; *p* < 0.0001) (Figure 1A,B). The excessive accumulation of collagen in the ECM of M-side was supported by the increased gene expression of collagen type I (measured by the procollagen type I structural component, encoded by the *COL1A1* gene (Figure 1C).

### 2.2. Increased Crosslinking in the Medial Part of Clubfoot

**Figure 2:**
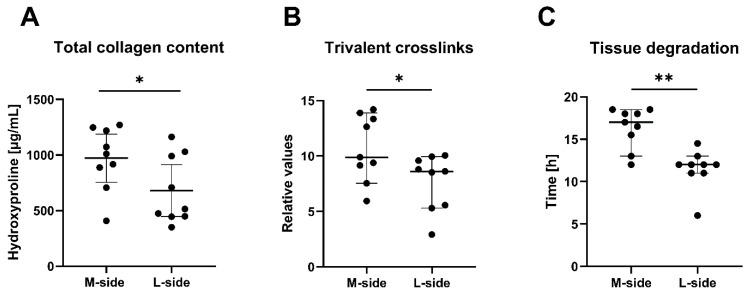
(**A**) Total collagen content measured by a hydroxyproline assay. (**B**) Level of trivalent collagen crosslinks in clubfoot tissue. The concentration of pyridinoline and deoxypyridinoline crosslinks obtained by ELISA was normalized to the collagen content of the same sample measured in parallel. (**C**) Resistance of clubfoot tissue to degradation by bacterial collagenase. The data in all graphs are presented as the median with a 95% confidence interval. Paired *t*-test; statistical significance: * *p* = 0.0199, * *p* = 0.0147, ** *p* < 0.0039 (respectively).

We determined the total amount of trivalent-pyridinoline and deoxypyridinoline crosslinks formed in collagens of M-side and L-side samples by an enzyme-linked immunosorbent assay (ELISA). The number of crosslinks in each sample was normalized to the collagen content of the sample measured in parallel (Figure 2A). We found significantly increased crosslinking in the M-side tissue compared to the L-side tissue (Figure 2B). The ELISA method does not distinguish between collagen types. This increase therefore involves crosslinks formed in all collagen types in the sample. However, most of the crosslinks can be attributed to those formed in type I collagen, which is the most abundant collagen type in clubfoot tissue.

We also evaluated the resistance of the tissue to degradation of collagen fibrils by a bacterial collagenase. We performed a degradation assay to test whether the increased crosslinking hinders collagen degradation by collagenases, and thus whether it can lead to unwanted collagen accumulation. We monitored the time needed for the complete degradation of the M-side and L-side tissue samples by bacterial collagenase type III, i.e., an enzyme with typical collagenase activity but with low secondary protease activity. Our data showed that the degradation of the M-side samples is significantly delayed in comparison to the time needed for the complete degradation of the L-side (Figure 2C).

### 2.3. BAPN (Beta-Aminopropionitrile) Is Safe for Clubfoot-Derived Cells

We isolated, propagated, and characterized cells from the contracted medial side of clubfoot tissue, which we have described in detail [21]. Briefly, the culture contained 96% of fibroblasts, with an admixture of myofibroblasts and fibrocytes (less than 3%), vascular smooth muscle (less than 0.1%), but no endothelial cells. In view of the decreasing proportion of myofibroblasts in the culture in later passages, we used only the first, second, or third passage for the experiments.

**Figure 3:**
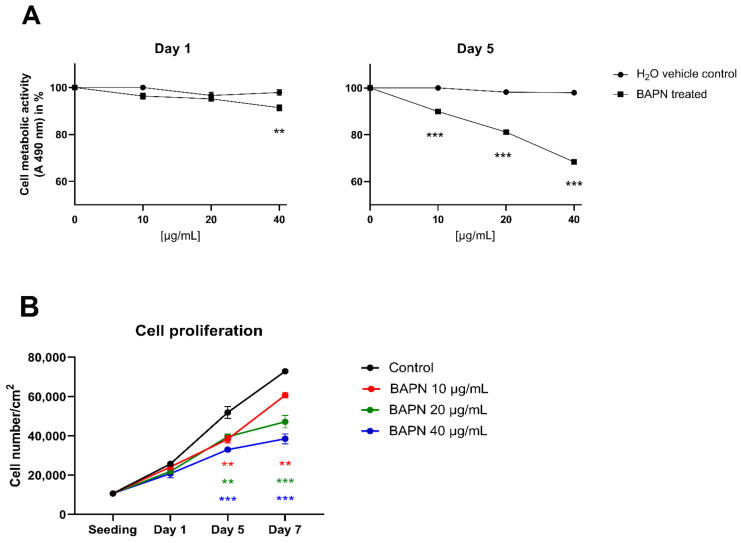
(**A**) Dose–response relationship effect of β-aminopropionitrile (BAPN) concentration on cell proliferation and viability, expressed as the cell metabolic activity after 1, 5, and 7 days of treatment: Points represent the percentage value of the metabolic activity of cells treated with different concentrations of BAPN or with H_2_O vehicle control, in comparison with the untreated control (regarded as the 100% value). (**B**) The effect of BAPN concentration on the cell proliferation rate. The data in all graphs are presented as mean ± SEM. One Way ANOVA, Dunnett’s test; statistical significance compared to the control: ** *p* < 0.01, *** *p* < 0.001.

We tested the direct cytotoxic effect of BAPN (10–40 µg/mL) on the viability of clubfoot-derived cells by an MTS metabolic assay together with a growth curve analysis. We interpreted the results of the MTS assay according to the International Standard for testing in vitro cytotoxicity [27] as an assessment of cell viability after 24 h of cultivation with the tested substance. According to the ISO standard, an agent that reduces cell viability to less than 70% of an untreated control sample is considered to have a cytotoxic potential. After 1 day of treatment, none of the concentrations of BAPN reduced the viability/metabolic activity of the cells to less than 70% of the control samples (Figure 3A). However, the concentration of 40 µg/mL of BAPN showed a statistically significant decrease in absorbance at 490 nm (A490) compared to control, but this decrease (to 91.4%) can be considered biologically non-significant, given the ISO limit. Beyond the ISO limits, we also evaluated the viability of the cells on day 5, where all tested concentrations of BAPN (10, 20, 40 µg/mL) reduced the metabolic activity of the cells significantly compared to controls (to 89.9%, 81.1%, and 68.4%, respectively); however, only the highest concentration reduced the activity to below 70% (Figure 3A).

In addition, we observed concentration-dependent inhibition of cell proliferation in the presence of BAPN (10–40 µg/mL) in comparison with the control cells. This inhibition reached statistical significance on day 5 and continued on day 7 of cell culture (Figure 3B).

### 2.4. BAPN inhibits Collagen Deposition and Crosslinking in Clubfoot-Derived Cells

**Figure 4:**
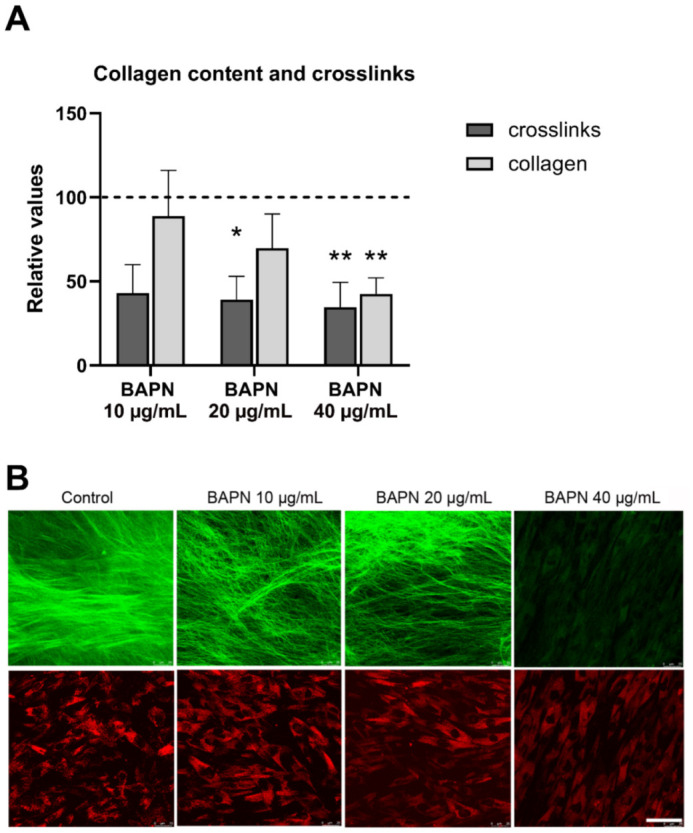
(**A**) Beta-aminopropionitrile (BAPN) reduced collagen crosslinking and insoluble collagen deposition into ECM after 3 weeks in culture. Both the amount of collagen deposited into the ECM and the concentration of the pyridinoline and deoxypyridinoline crosslinks were normalized to the total protein content in the sample. Data presented as mean ± SD. Kruskal–Wallis ANOVA, Dunn’s test; statistical significance: * *p* = 0.0252, ** *p* = 0.0028. (**B**) Second harmonic generation (SHG) imaging of collagen type I fibers after 3 weeks in culture. The decrease in the SHG signal depicted in green pseudocolor (upper panel) demonstrates the effect of BAPN on the correct structural assembly of collagen type I. The autofluorescence of the fibroblasts is depicted in red pseudocolor (lower panel). Leica DMi8 confocal microscope, obj. 63× water immersion, Scale bar = 50µm.

After three weeks of culture, we assessed the direct effect of BAPN on collagen crosslinking as well as its effect on the deposition of insoluble collagen into ECM in vitro. BAPN at 20 µg/mL and at 40 µg/mL significantly reduced the amount of pyridinoline and deoxypyridinoline crosslinks; however, only the highest concentration (40 µg/mL) led to a significant decrease in deposited collagen, as measured by a hydroxyproline assay (Figure 4A). The effect of BAPN on the correct structural assembly of collagen type I fibers in the ECM was evaluated by generating a second harmonic (SHG) signal visualized under a confocal microscope. A generally weaker signal in comparison with the untreated control was observed in all BAPN-treated samples, and a markedly weaker signal was obtained in samples treated with 40 µg/mL of BAPN (Figure 4B).

### 2.5. Effect of BAPN on Tissue-Like Contraction of ECM in a 3D In Vitro Model

**Figure 5:**
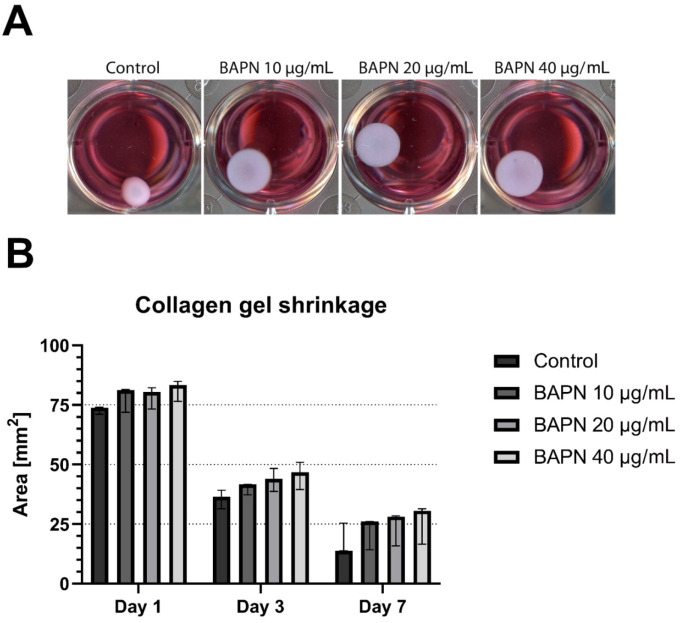
Contraction of 3D cell-populated collagen gel lattices: (**A**) Representative images taken on day 7, and (**B**) an analysis of changes in the collagen gel areas during the course of β-aminopropionitrile (BAPN) treatment. Data presented as the median with a 95% confidence interval. One Way ANOVA or Kruskal–Wallis ANOVA; statistical significance in comparison with the control: not significant at any time point.

Apart from the collagen accumulation, another important aspect of the potential use of BAPN as a therapeutic is its ability to reduce the contraction of ECM. In a 7-day experiment, we monitored the tissue-like contraction of collagen gel lattices populated with clubfoot-derived cells in the presence of BAPN (Figure 5). In both the BAPN-free control and BAPN-treated samples, the collagen lattices underwent cell-mediated shrinkage, which progressed almost uniformly from day 1 to 7. However, BAPN attenuated this shrinkage in a concentration-dependent manner. An increasing concentration of BAPN resulted in a gradual reduction in the lattice contraction—BAPN 40 µg/mL elicited the greatest reduction. This trend was observable already on day 1, after the administration of BAPN, and became clearly apparent on day 3. On day 7, after seeding, all three tested concentrations of BAPN considerably reduced the contraction of the collagen lattice in comparison with the untreated control samples. Although these differences were evident (Figure 5A), they were not sufficiently large enough to establish statistical significance at any measured time interval (Figure 5B).

## 3. Discussion

Correcting complex deformities such as relapsed clubfoot remains a long-term challenge, especially in older children and in individuals who do not fully adhere to the Ponseti treatment regimen. However, no pharmacological strategies aimed at supporting and facilitating conservative treatment are being developed to replace the surgical approaches used in problematic resistant cases of this kind.

Our previous research [16,17,18] and studies reported by other authors [11,12,13,14] have shown the presence of profibrotic proteins and myofibroblasts in the contracted tissue of clubfoot. In our study, we have focused on a quantitative analysis of collagen type I and, in particular, collagen crosslinking as a possible cause of the increased tissue rigidity, which may impede correction with the use of the Ponseti method. We analyzed stiff, contracted tissue from the medial side of clubfoot together with non-contracted tissue from the lateral side of clubfoot. For the first time, we have identified a significantly increased expression of the *COL1A1* gene and a significantly increased content of type I collagen in the medial side tissue (Figure 1). Both we other researchers have already confirmed the upregulation of type III, V, and VI collagen in the M-side [17,20]. An increased content of fibrillar collagens (type I, III, and V) is typically associated with fibrosis; in some fibrotic disorders, the ratio of collagen types reflects the stage of fibrosis (reviewed in [28]). The upregulated transforming growth factor beta (TGF-β1) and transforming growth factor-inducible protein (TGFBIP) found earlier [17,20], together with an activated beta-catenin signaling pathway [20], presumably promote fibrogenesis and drive fibroblasts into the synthetic and contractile phenotype of myofibroblasts. Collagen type VI is a non-fibrillar collagen interconnecting the individual fibrils of collagen type I and III, and thus stabilizing the collagen network [28]. In Dupuytren’s disease, collagen type VI was proved to maintain the myofibroblast phenotype [29].

We have also identified an increased level of trivalent pyridinoline and deoxypyridinoline crosslinks, which stabilize collagen fibrils (Figure 2B). This is in contradiction with the findings reported by Van der Sluijs and Pruys [30], who carried out the only study that has so far been presented on this subject. They found no increase in crosslinking in the 21 clubfeet of various types (predominantly idiopathic, but also syndromal, e.g. associated with spina bifida). These authors analyzed the tissue of the posterior joint capsule of clubfoot, which may have delivered different results; however, it is probable that this difference arose from the interpretation in the absence of controls in their study. They compared the number of crosslinks with the standard level of crosslinks in normal adult tendon tissue described in the literature, although it has been proven that crosslinking increases with age [31,32]. The collagen content (0.16 mg/mg of wet tissue) in Van der Sluijs’s study is referred to as normal, without any direct comparison or citation. Van der Sluijs and Pruys [30] also investigated the association of changes in collagen properties (pyridinoline and deoxypyridinoline crosslinks and alignment of collagen fibers), with the clinical Dimeglio score used to describe clubfoot severity. They found no correlation between the measured parameters of clinical stiffness, type of clubfoot, or the gender or age of the patients. Because we had a limited number of patients in our crosslinking analysis (*n* = 9) and a generally quite specific set of patients (i.e., with resistant relapsed clubfeet) we did not perform a correlation analysis between crosslinks and patients’ clinical data.

Increased crosslinking has been implicated in various fibrotic diseases, and seems to be a general fibrotic phenomenon [33,34,35,36]. There are strong indications that crosslinks (especially the pyridinoline type) reduce the susceptibility of collagen to proteolytic degradation, and, in this way, the crosslinks contribute to the unwanted collagen accumulation in fibrosis [33,34,37]. This is in agreement with our data in all key points: we have proved (i) an elevated amount of crosslinking (Figure 2B); (ii) delayed degradation of ECM by collagenase (Figure 2C); (iii) increased accumulation of collagen type I in the tissue on the medial side of clubfoot (Figure 1). Generally, these changes are known to contribute to increased tissue stiffness during the development of fibrosis. The difference in stiffness between the M-side and L-side tissues of clubfoot is evident when the samples are excised during surgery and when they are processed during cell isolation.

Collagen types, collagen fiber orientation or thickness, as well as the collagen crosslinks and the number of crosslinks, are strongly affected by the amount and type of mechanical stress, in addition to other factors (e.g., tissue type or diet) [38]. The arrangement of collagen fibers differs from parallel fibers in tendons to orthogonal grids in the cornea [39]. The specific orientation is sometimes also observed in pathological cases. Rodriguez et al. [40] reported strong anisotropy of the fibers in the fibrotic contracture cords of Dupuytren’s disease. On the other hand, there is no directional preference of collagen fibers in the immunohistochemical (IHC) M-side sections in our study, in agreement with [11]. The strong isotropy in IHC seems to reflect the multiaxial movement, i.e., the multiaxial force load as the collagen fibers are naturally assembled primarily to withstand the tension load. To provide structural support for the requirements of the tissue for strength in a specific load direction, the tissue controls the level of collagen molecule crosslinking [39]. However, extensive crosslinking can lead to an increase in tissue stiffness, and the tissue can become more brittle, or it can lead to a reduction in tissue remodeling and collagen turnover [41].

Increased collagen crosslinking in other fibroproliferative diseases is executed by dysregulated enzymatic post-translational modifications of collagen. The way in which the crosslinks are formed affects the topology of the collagen molecule, which in turn affects the mechanical properties of the collagen matrix in general. For example, upregulated lysyl hydroxylase 2 (LH2), an enzyme responsible for the amount of telopeptidic hydroxylysine formation, affects the quality of the collagen crosslinks in hypertrophic scars and in Dupuytren’s disease [42] (reviewed in [36]). In contrast to LH2 enzyme, posttranslational modifications by lysyl oxidase enzymes (LOXs) occur at a later point in collagen biosynthesis, though they are greatly affected by the previous action of LH2. The expression and the activity of LOX affects the quantity of crosslinks formed by catalyzing aldehyde formation in telopeptides, stabilizing the collagen triple helices into a fiber by intermolecular bridges [43]. Increased levels of LOX have been found in the tissue fibrosis of various organs with excessive remodeling (e.g., in mice after myocardial infarction [26]; in patients with systemic sclerosis [44]; and in patients with idiopathic pulmonary fibrosis [45]).

The observed changes in collagen production, deposition, and processing in clubfoot tissue, together with a knowledge of the pathogenesis of more extensively studied fibroproliferative diseases, could form a basis for the development of future targeted treatment strategies. We have recently demonstrated that inhibiting LH2 by Minoxidil in clubfoot-derived cells leads to decreased short-term and long-term levels of deposited collagen, together with reduced tissue-like contraction in a 3D collagen gel model [21]. To further explore the idea in this study, we tested the therapeutic potential of β-aminopropionitrile (BAPN), an agent that reduces collagen crosslinking by inhibiting members of the LOX family on fibroblast-like cells isolated from clubfoot medial side tissue.

In the course of a 5-day experiment, we observed a dose-dependent effect of BAPN slowing cell proliferation (Figure 3). However, the concentrations of 10–40 µg/mL of BAPN were not directly cytotoxic for clubfoot-derived cells, and they can be considered safe for use in vitro. BAPN administered at 20 µg/mL and at 40 µg/mL significantly reduced crosslinking in 2D cell culture (Figure 4A), and BAPN at 40 µg/mL reduced collagen deposition into ECM (Figure 4B).

**Figure A1:**
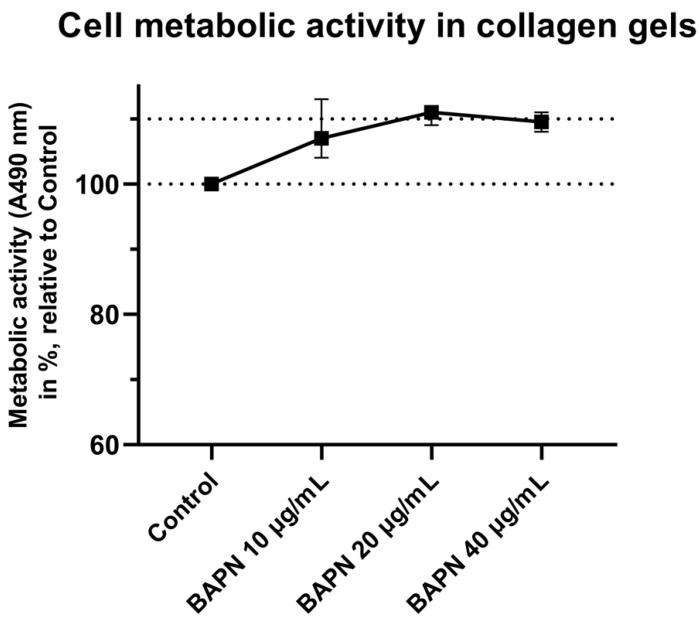
Viability of cells embedded in collagen gel lattices after 7 days of treatment with 0–40 µg/mL BAPN, represented by cell metabolic activity as measured by an MTS assay. Kruskal–Wallis ANOVA; statistical significance compared to the control: not significant.

For a study of the cell-mediated contraction of ECM, we used 3D cell-populated collagen gel lattices. Cell-induced contraction of ECM plays an important role in fibrosis, which indicates that mechanical forces contribute to the development and progression of the fibrosis. Collagen 3D gel lattices are a useful tool for evaluating the collagen–cell interaction and tissue remodeling in vitro. In our experiments, the contraction that occurred during the first two days was probably only partially dependent on the anti-crosslinking effects of BAPN. Within this time period, the traction forces of the cells are largely affected by changes in cell morphology, spreading, and proliferation [46], rather than by the synthesis and processing of collagen. Starting on day 3, the shrinkage of the gels was gradually inhibited by an increasing concentration of BAPN, establishing a clear concentration-dependent trend. Unfortunately, the effect of BAPN was not significant, even at a later time interval (day 7), as the data suffered from large variability (Figure 5). However, the viability of all cells was comparable on day 7 (Appendix A, Figure A1), which suggests that the reduction in gel contraction should not be attributed to any cytotoxic effects of BAPN. The mechanism and the degree of gel shrinkage is dependent on many considerations (attached vs. floating gels, cell population density, collagen origin and concentration, presence of fetal serum, etc.). It is therefore difficult to compare the results of contraction assays performed by different authors. Woodley et al. [47] showed that the telopeptide domains involved in collagen crosslinking are necessary for the in vitro gel contraction. This implies that BAPN mediated the inhibition of LOX enzymes, catalyzing the formation of telopeptidic crosslinks, and might be responsible for the reduced contraction of the gel. We did not measure the stiffness of the collagen gels after they had been treated with BAPN, though this issue has been investigated in vivo by many authors. For example, Oxlund et al. [19] demonstrated that the stiffness of the femoral bones of BAPN-injected rats decreased by 30% in comparison with control rats, measured by the Young’s modulus of elasticity. Kato et al. [48] reported decreased stiffness of the left ventricle chamber and myocardium in pigs treated with BAPN.

The values describing the collagen gel shrinkage at later time intervals are variable, but a clear trend of a favorable concentration-dependent effect of BAPN can be observed in our study. We therefore believe that it would be beneficial if the effect of BAPN on the contraction of the collagen lattice could be verified in an additional set of experiments or, better still, with the use of a more sophisticated ECM model.

It can be concluded that we have demonstrated an increased content and expression of collagen type I, as well as an increased formation of trivalent collagen crosslinks, and a higher resistance to proteolytic degradation in the stiff, contracted tissue of the clubfoot medial side. We have determined safe concentrations of LOX inhibitor BAPN (10–40 µg/mL) for use in a clubfoot-derived cell culture. We have observed that concentrations of 20 and 40 µg/mL significantly reduced collagen crosslinking in cell culture. Administering 40 µg/mL of BAPN to the cell culture also decreased the collagen deposition into ECM, and exhibited the greatest reduction in the tissue-like contraction of a 3D collagen gel lattice model. Based on these data, we hypothesize that BAPN, or a substance with a similar effect, could be relevant for the design of a potential anti-fibrotic therapy to accompany the standard treatment for relapsed and resistant clubfoot.

When considering a possible adjunctive pharmacological treatment for clubfoot, the goal should be to prevent an abnormal accumulation of highly crosslinked collagen, but not to significantly reduce collagen deposition per se, as collagen deposition is a part of the remodeling process occurring in the affected tissue. In future studies, it is therefore necessary to test the optimal concentrations of BAPN again in an appropriate and well-defined model of clubfoot, e.g., a more sophisticated 3D in vitro model or a newly-developed animal model, and with an anti-fibrotic substance incorporated, e.g., into a macromolecular carrier with gradual drug release. Pharmacological inhibition of collagen crosslinking on the level of LOXs or other enzymes involved could be a promising way to reverse local fibrosis in clubfoot by changing the ECM properties towards the easier degradability of collagen.

## 4. Materials and Methods

### 4.1. Biological Material

Samples from a total of 22 patients (2 females, 20 males; mean age 53.09 months, SD = 24.71) were obtained during surgery for idiopathic clubfeet relapsed after unsuccessful Ponseti treatment. Detailed information is provided in Appendix A, Table A1. Two samples were obtained from each patient: contracted medial (M-)side tissue localized between the medial malleolus, sustentaculum tali and the navicular bone, and non-contracted lateral (L-)side tissue from the surface of the calcaneocuboid joint. Due to the samples being a dense irregular connective tissue, no body axis information is given. The samples were carefully divided, when possible, with regard to the size of the samples. Sets of corresponding M-side and L-side samples were processed: 10 samples were used for immunohistochemistry, 9 samples were used for collagenase digestion experiments and crosslinking analysis, 7 samples were processed for PCR analysis, and 5 samples were used for establishing primary cell cultures. Due to sample size and the specific storage conditions necessary for different analyses, samples from all patients could not be used in all experiments.

The study was conducted in accordance with the principles of the Declaration of Helsinki and with the Institutional Ethics Committee’s approval. The parents or legal guardians of all patients provided informed written consent to participate. This study is analytical, prospective, level of evidence IIB.

### 4.2. Immunohistochemistry

Tissue samples (M-side and L-side sets) from 10 patients (1 female, 9 males, mean age 47.40 months, SD = 18.30) were fixed in Baker’s solution, embedded in paraffin, and cut in a microtome into serial sections 5 µm in thickness. Collagen fibers in these samples were identified using a primary antibody for collagen type I (Cat. No. LSL-LB-1197, Rabbit polyclonal, dilution 1:800 in PBS, CosmoBio, Co. Ltd., Tokyo, Japan), incubation for 1 h at room temperature (RT). Antigen retrieval, hydrogen peroxide block, protein block, secondary antibody reaction, and visualization were then performed according to the Abcam protocol by applying the EXPOSE Mouse and Rabbit Specific HRP/DAB IHC Detection Kit (ab236466, Abcam, Cambridge, UK). The samples were counterstained with hematoxylin. Positive staining of the investigated antigen was evaluated by light microscopy, and quantification was performed using image analyzer signal thresholding (Nikon Imaging Software NIS-Elements 3.0 AR, Laboratory Imaging, Prague, Czech Republic). The intensity of the signal emanating from 10 different areas of each sample was detected, and the percentage of the area showing positive immunostaining was calculated.

### 4.3. Total mRNA Isolation and Real-Time PCR

Real-time PCR was performed to assess differences in the relative mRNA expression of collagen type I in M-side and L-side tissue. Samples were taken from the M side and L-side tissue sets of 7 patients (1 female, 6 males; mean age 47.42 months, SD = 37.11). From each of these tissue samples, three pieces (20 mg each) were taken for total mRNA isolation per patient (representing *n* = 1). Briefly, the tissue samples were homogenized with MagNA Lyser Green Beads (Roche, Basel, Switzerland), and the isolation procedure was performed with the Animal Tissue RNA Purification Kit (Norgen Biotek, Thorold, Canada) following the same protocol with the use of the same instruments, as described in Novotny et al. [18]. RNA in a concentration of 1 µg/mL was then used for reverse transcription into cDNA, using the Omniscript Reverse Transcription Kit (205113; Qiagen, Hidlen, Germany) and Random Primer Mix (New England BioLabs, USA). Real-time PCR was performed using 5xHOT FIREPol Probe qPCR Mix Plus (ROX) (08–14-00008, Solis BioDyne, Tartu, Estonia) and TaqMan Gene Expression Assays (Thermo Fisher Scientific, Waltham, MA, USA) labeled with FAM reporter dye specific to human collagen type 1 alpha-1 chain (*COL1A1*; Hs00164004_m1) and a reference gene for Beta-2-microglobulin (*B2M*; Hs00187842_m1). A final reaction was conducted using the Viia 7 Real-time PCR System (Applied Biosystems, ThermoFisher Scientific, Waltham, MA, USA) in a MicroAmp Fast Optical 96-well reaction plate (Applied Biosystems, ThermoFisher Scientific, Waltham, MA, USA). The total reaction volume was 20 μL, and the cycle parameters were as follows: incubation at 95 °C (10 min), followed by 40 cycles of 95 °C (15 sec) and 60°C (1 min). The results are analyzed and presented as ΔCt values for each patient.

### 4.4. Collagenase Digestion Assay and Analysis of Collagen Crosslinks in the Tissue

Tissue samples (M-side and L-side sets) from 9 patients (1 female, 8 males; mean age 52.88 months, SD = 21.64) were subjected to a defined collagenase treatment (300 U/mL of clostridial collagenase type III, dissolved in DMEM; Worthington Biochemical Corp., Lakewood, NJ, USA), 250 µL of enzyme solution per 10 mg of tissue. The samples were incubated at 37 °C with mild vortexing until they completely degraded into small pieces. After collagenase digestion treatment, the samples were lyophilized and were weighed. The samples were then proteolytically digested using acid hydrolysis (50 µL of 6N HCl, 105 °C overnight) and were neutralized (50 µL of 6M NaOH). The concentration of pyridinoline and deoxypyridinoline crosslinks was measured by ELISA, using the MicroVue Serum PYD Enzyme Immunoassay kit (Cat. No. 8019, MicroVue, Quidel, San Diego, CA, USA). The concentration of crosslinks was normalized to the collagen content of the same sample measured in parallel by a hydroxyproline colorimetric assay (K555, BioVision, Milpitas, CA, USA), according to the manufacturer’s instructions.

### 4.5. Isolation and Cultivation of Cells

Tissue samples (M-side and L-side sets) from 5 patients (1 female, 4 males; mean age 43.20 months, SD = 24.40) were mechanically minced and enzymatically digested to isolate primary cell cultures, which were then propagated and characterized, as described previously [21]. For the experiments, the cells were incubated in DMEM medium (Sigma–Aldrich, Burlington, MA, USA) containing 10% of fetal bovine serum (FBS; Gibco, ThermoFisher Scientific, Waltham, MA, USA) with the addition of 0, 10, 20, and 40 µg/mL of BAPN (beta-aminopropionitrile, CDS007521, Sigma–Aldrich, Burlington, MA, USA; reconstituted in sterile dH2O to 5 mg/mL stock solution). The media were changed every 3–4 days. Only the cells from contracted M-side tissue were used in the experiments with BAPN. A vehicle equivalent control to BAPN (corresponding volume concentration of dH2O) was tested in all experiments and was found not to be statistically significant.

### 4.6. Cytotoxicity Testing

The cells were seeded into a 24-well plate at an initial density of 2 × 104 cells/well (10,730 cells/cm^2^) in 1 mL of DMEM with 20% of FBS. After 24 h, the cells were serum-starved in DMEM with 1% of FBS for another 24 h to synchronize the cell cycle. Afterwards, the cells were grown in DMEM containing 10% of FBS with 0, 10, 20, and 40 µg/mL of BAPN for 3, 5, and 7 days. The viability of the cells in the presence of BAPN was estimated by a standard metabolic MTS colorimetric assay (G3582, CellTiter 96 AQueous One Solution Cell Proliferation Assay, MTS; Promega Corp., Madison, WI, USA) according to the manufacturer’s instructions. The reduction in MTS was measured at 490 nm (with a reference wavelength at 650 nm) using the VersaMax Absorbance Microplate Reader (Molecular Devices, San Jose, CA, USA). Subsequently, the cells were fixed in ice-cold 70% ethanol and were stained for 1 h (RT, in the dark) with Texas Red C2-Maleimide (20 ng/mL in PBS; stains the cell membrane and cytoplasmic proteins in red color) together with Hoechst 33258 (5 μg/mL in PBS; stains nuclei in blue color) for an evaluation of the cell proliferation. Microphotographs (15 per sample per well) of randomly chosen fields were taken with an Olympus IX51 epifluorescence microscope, equipped with a DP70 camera (both Olympus Corp., Tokyo, Japan). The microphotographs were analyzed in ImageJ FIJI software (https://imagej.net/Fiji; Accessed 1 July 2020 [49]). The cell population densities on the samples were calculated and are presented as cell numbers per cm^2^.

### 4.7. Collagen Deposition and Crosslinking in Cell Culture with/without BAPN

The cells were seeded into 12-well plates at an initial density of 6 × 104 cells/well and were incubated in the presence of 0, 10, 20, and 40 µg/mL of BAPN in DMEM containing 10% FBS and 50 µg/mL of L-ascorbic acid 2-phosphate trisodium salt (49752, Sigma–Aldrich, Burlington, MA, USA) for 3 weeks. The cells were lysed in RIPA buffer (R0278, Sigma–Aldrich, Burlington, MA, USA), and a Pierce BCA assay (Cat.No. 23225, ThermoFisher Scientific, Waltham, MA, USA) was performed according to the manufacturer’s instructions to measure the total protein content. The samples were then proteolytically digested using acid hydrolysis (50 µL of 6N HCl, 105 °C overnight) and were evaporated. The amount of collagen deposited into the extracellular matrix was quantified by the Hydroxyproline Colorimetric Assay kit (K555, BioVision, Milpitas, CA, USA), according to the manufacturer’s instructions, and the absorbance was measured using a VersaMax Absorbance Microplate Reader (Molecular Devices, San Jose, CA, USA) at 560 nm. The amount of deposited collagen was normalized to the total protein content, which was measured by a Pierce BCA assay (Thermo Fisher Scientific, Waltham, MA, USA). The concentration of pyridinoline and deoxypyridinoline crosslinks was measured by ELISA using the MicroVue PYD Enzyme Immunoassay kit (Cat. No. 8010, MicroVue Quidel, San Diego, CA, USA). The concentration of crosslinks was normalized to the total protein concentration in the same sample.

### 4.8. Second Harmonic Generation (SHG) Imaging of Collagen Type I

The cells were seeded on microscopic glass coverslips in a 24-well plate, were cultivated for 3 weeks, and were visualized using a 63× water immersion objective (HC PL APO CS 2 63×/1.20 Water), mounted on a Leica DMi8 inverted microscope with a Leica TCS SP8 X confocal unit (Leica Microsystems, Weltzar, Germany). A Chameleon Discovery TPC pulsed femtosecond laser (Coherent Inc., USA; 860 nm tunable output, 80 Mhz, 1.6 W) was used to generate SHG and autofluorescence signals. Leica non-descanned HyD detectors and a 430/24 band-pass filter were used for collecting SHG signals, and Leica non-descanned HyD detectors and a 610/75 band-pass filter were used for collecting autofluorescence signals.

### 4.9. Cell-Mediated Collagen Contraction Assay

A three-dimensional (3D) in vitro collagen model of ECM (CytoSelect 24-Well Cell Contraction Assay Kit—floating matrix model, CBA-5020, Cell Biolabs, San Diego, CA, USA) was used to describe the potential of BAPN to reduce ECM contractility. Collagen gel lattices were prepared according to the manufacturer’s instructions, with 193 × 103 cells mixed into 0.5 mL of collagen gel per well, and were incubated in DMEM with 10% of FBS and either BAPN (10, 20, 40 µg/mL) or the maximum concentration of dH_2_O (vehicle control). Gels without cells were used as a negative control. Changes in the sizes of the gel lattices were monitored for 7 days. Images of the well plate with gel lattices were acquired using an EPSON V500 flatbed scanner (Seiko Epson, Suwa, Japan) and were analyzed in ImageJ FIJI software.

### 4.10. Statistical Analysis

Statistical analyses and data visualization were performed in GraphPad Prism 9 (GraphPad Software, San Diego, CA, USA). Normality was checked by the Shapiro–Wilk test. Paired two-tailed t-tests were performed on the data from all experiments to evaluate the differences between M-side and L-side tissues, with the exception of Real-time PCR and the collagenase digestion assay, for which the Wilcoxon matched-pairs test was used. Data from the evaluations of the tissues (immunohistochemistry, qPCR, ELISA, tissue degradation assay) are presented as the median with a 95% confidence interval. One Way Analysis of Variance (ANOVA) with the Dunn’s test or Kruskal–Wallis ANOVA with the Dunnett’s post hoc test were performed on the experimental data to evaluate the effects of different concentrations of BAPN on clubfoot-derived cells. Data from the in vitro evaluations are presented as: mean ± SEM (cell proliferation and metabolic assay), mean ± SD (collagen crosslinking and collagen deposition into ECM), or median with a 95% confidence interval (collagen gel lattice contraction assay). Significance of the differences was set at *p* < 0.05.

## Data Availability

The datasets generated during and/or analysed during the current study are available from the corresponding author on reasonable request.

## Appendix A

**Table A1:** Detailed data of 22 patients (sample donors) with a relapse of idiopathic congenital clubfoot: The parents of these patients were generally non-compliant with the Ponseti regime, or were unable to maintain proper treatment by applying an abduction bar, or even failed to come to scheduled examinations. Abbreviations: M (male), F (female) AT (Achilles Tendon Tenotomy), AT+R (Achilles Tendon Tenotomy + Retenotomy), MK (McKay), Fw (Fowler), N (No), Y (Yes), PR (Posteromedial Release).

Patient	Gender	Dimeglio Clubfoot Classification	Age During Sample Acquisition	Ponseti Casting	Type of Surgery	Previus Surgeries	Clubfoot Family Anamnesis
1	M	III	75	5	PR	AT	N
2	M	III	34	6	PR	AT+R	N
3	F	III	16	5	MK	AT	N
4	M	IV	54	8	PR	AT	N
5	M	IV	32	unknown	MK	N	N
6	M	IV	32	unknown	MK	N	N
7	M	IV	54	6	PR	AT	Y
8	M	IV	68	6	PR	AT	N
9	M	IV	55	6	PR	AT	N
10	M	III	60	6	PR	AT	N
11	M	III	44	10	PR	AT	N
12	M	IV	54	6	PR	AT	Y
13	M	III	68	7	PR	AT	N
14	M	III	38	10	PR	AT	N
15	M	IV	66	6	PR	AT	N
16	M	III	84	5	MK	AT	Y
17	F	IV	22	10	MK	AT+R	N
18	M	IV	12	10	MK	N	N
19	M	III	60	5	MK	N	Y
20	M	III	124	5	MK	AT	N
21	M	III	58	6	PR	AT	N
22	M	III	58	3	Fw	AT	N
Patients total: 22	2 × F 20 × M	12 × III 10 × IV	Mean 53.09 months (SD = 24.71)	Mean 6.55 (SD = 2.01)	8 × MK 13 × PR 1 × Fw	18/22	4/22
